# Complete Defluorination of Perfluorinated Compounds by Hydrated Electrons Generated from 3-Indole-acetic-acid in Organomodified Montmorillonite

**DOI:** 10.1038/srep32949

**Published:** 2016-09-09

**Authors:** Haoting Tian, Juan Gao, Hui Li, Stephen A. Boyd, Cheng Gu

**Affiliations:** 1 State Key Laboratory of Pollution Control and Resource Reuse, School of the Environment, Nanjing University, Nanjing 210023, P. R. China; 2 Key Laboratory of Soil Environment and Pollution Remediation, Institute of Soil Science, Chinese Academy of Sciences, Nanjing, Jiangsu 210008, P. R. China; 3 Department of Plant, Soil and Microbial Sciences, Michigan State University, East Lansing, Michigan 48824, United States

## Abstract

Here we describe a unique process that achieves complete defluorination and decomposition of perfluorinated compounds (PFCs) which comprise one of the most recalcitrant and widely distributed classes of toxic pollutant chemicals found in natural environments. Photogenerated hydrated electrons derived from 3-indole-acetic-acid within an organomodified clay induce the reductive defluorination of co-sorbed PFCs. The process proceeds to completion within a few hours under mild reaction conditions. The organomontmorillonite clay promotes the formation of highly reactive hydrated electrons by stabilizing indole radical cations formed upon photolysis, and prevents their deactivation by reaction with protons or oxygen. In the constrained interlayer regions of the clay, hydrated electrons and co-sorbed PFCs are brought in close proximity thereby increasing the probability of reaction. This novel green chemistry provides the basis for *in situ* and *ex situ* technologies to treat one of the most troublesome, recalcitrant and ubiquitous classes of environmental contaminants, i.e., PFCs, utilizing innocuous reagents, naturally occurring materials and mild reaction conditions.

Perfluorinated compounds (PFCs) comprise a large group of manufactured chemicals, including the common commercial products Teflon^TM^, Stainmaster^TM^, Scotchguard^TM^, used as coatings to repel both water and oil, and to prevent soiling and adhesion; they also have many special applications such as fire-fighting foams^1^,^2^,^3^,^4^. In the past half-century, PFCs have been produced in large quantities, used in many household applications, and disposed of with little or no regulation. Perfluorinated chemicals are highly persistent in the environment, bioaccumulative, and toxic. There is widespread and well documented exposure of humans and wildlife to PFCs^5^,^6^,^7^,^8^, most notably perfluooctanoic acid (PFOA) and perfluorooctane sulfonate (PFOS) which are the common byproducts of the primary commercial products^5^. Prior research has indicated that PFOS is unable to be degraded under any known environmental conditions hence posing an ongoing threat to human and ecosystem health^6^,^7^. Related to their extraordinary persistence, PFCs are widely detected in rain water, ice caps, soils, air, surface waters and sewage sludges^2^. Human and wildlife exposure to PFCs is considered ubiquitous. The Centers for Disease Control report that PFCs are detected in humans worldwide, and that various modes of toxicity have been observed in animal models such as developmental and reproductive toxicity including neonatal mortality, cancers of the liver and bladder and several other toxicological endpoints^9^. The potential for direct exposure of the developing fetus is of great concern for PFCs, which is known to cross the placenta^10^. Human health risks across the entire structural class of PFCs continue to be assessed as a part of the National Toxicology Program^11^.

The C-F bond is recognized as the strongest covalent bond (531.5 kJ mol^−1^)^12^. This, combined with a high reduction potential (E_0_ = 3.6 V), results in the inherent structural stability of PFCs which resist chemical and biologically-mediated degradation^12^. While the terminal functional groups of PFCs (e.g. carboxylate and sulfonate) may be subject to cleavage, breaking the C-F bonds is far more difficult to achieve. Recently, photo-catalysis, electrochemical oxidation and sonochemical decomposition were used to degrade PFCs via defluorination. However, the photocatalytic defluorination of PFCs was incomplete, and toxic by-products were formed^13^,^14^. The short operating life of electrodes and the high concentrations of supporting electrolytes needed to ensure complete detoxification of PFCs also limits application of the electrochemical method for wastewater treatment^15^,^16^,^17^. Even ultrasonic degradation could completely mineralize PFOA and PFOS into CO, CO_2_, F^−^ and SO_4_^2−^ through the pyrolytic reaction as the transient collapse of cavitation bubbles, the degradation process was sensitive to changes in both ultrasonic (frequency and power) and experimental conditions (temperature and dissolved gas)^18^,^19^. Processes that achieve complete defluorination of PFCs under mild reaction conditions have until now remained elusive.

The hydrated electron is the most reactive reducing species known^20^, and hence is highly efficient in the reduction of PFCs^21^,^22^. In prior studies, hydrated electrons were generated by photoionization of iodide or sulfite ions^22^,^23^, which constrains the application of these reactions. In addition to the toxicity of iodide and sulfite, the reactions require alkaline and anoxic conditions to prevent the rapid consumption of hydrated electrons by protons or oxygen^22^,^23^. The comparison of experimental conditions for different treatments of PFCs is listed in Supplementary Table S1.

We recently demonstrated that hydrated electrons can be generated when indole or its derivatives are irradiated with sunlight, especially in the presence of the montmorillonite clay^24^. The clay mineral provides a planar negatively-charged surface that can stabilize indole radical cations, consequently inhibiting their recombination with hydrated electrons. Moreover, the photoreduction process is strongly dependent on the interaction between contaminants and clay mineral, higher adsorption increases the contact possibility of contaminants with hydrated electrons^24^. However, in our previous study, under natural conditions, the quantum yield of hydrated electrons is low and the reaction must occur in the absence of oxygen. The objective of this study is to develop a novel method to effectively degrade PFCs under mild reaction conditions. It has been reported that the photoionization efficiency of indole derivative, 3-indole-acetic-acid (IAA) is much higher than indole^25^ and the generation of hydrated electron increases sharply as the decrease of excitation wavelength^26^. Furthermore, previous studies showed that organic modification of montmorillonite could significantly enhanced the sorption of organic compounds^27^. Therefore, in this study, IAA, mercury lamp (254 nm) and organomodified montmorillonite were used instead of indole, sunlight and natural montmorillonite to significantly promote the production of hydrated electrons and the adsorption of PFCs on clay minerals. Here, we demonstrate the complete defluorination of both PFOA and PFOS by hydrated electrons induced from IAA in the interlayer region of organomontmorillonite under mild conditions. This novel reaction does not require anoxic alkaline conditions or toxic reagents, and hence obviates the constraints associated with prior reactions that attempt to use hydrated electrons to degrade PFCs.

## Results and Discussion

### Photodegradation of PFCs catalyzed by organoclay

**Figure 1:**
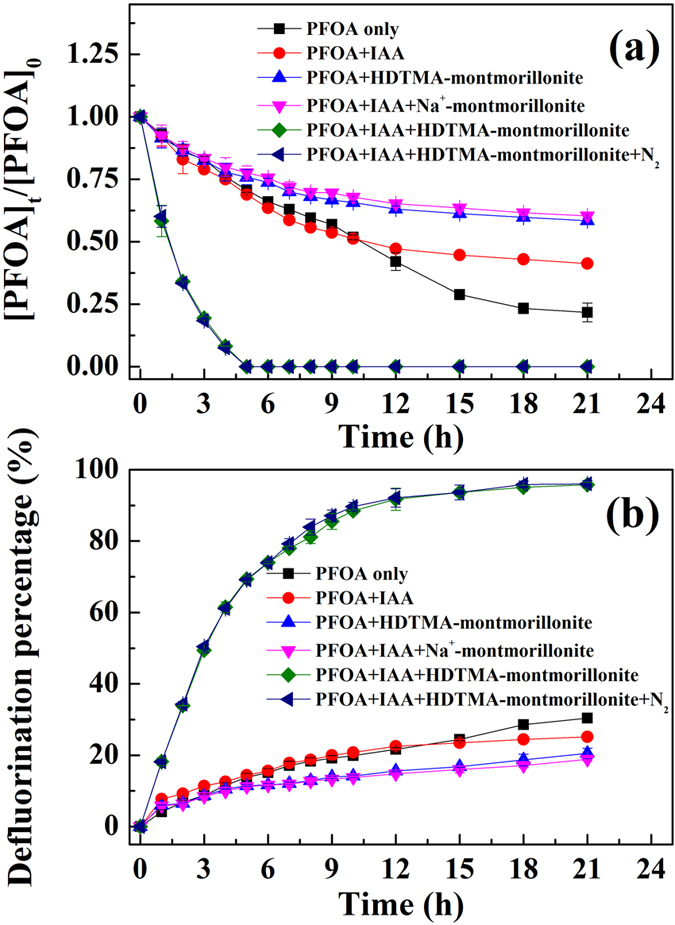
(**a**) Photodegradation and (**b**) defluorination of PFOA by 3-indole-acetic-acid under the irradiation of a mercury lamp as a function of time in the presence of HDTMA-montmorillonite. Experimental conditions: the initial concentrations of PFOA, IAA, and clay mineral were 10 mg L^−1^, 1 mM and 2.2 g L^−1^, respectively; pH was adjusted to 6.0 by adding NaOH and HCl; a 36 W low-pressure mercury lamp was used to provide light irradiation.

The photodegradation experiments of PFOA and PFOS were conducted by irradiation from a 36 W low-pressure mercury lamp with the maximum emission at 254 nm. The clay used was homoionic HDTMA-montmorillonite^27^. In direct photolysis of PFOA, about 50% degradation of PFOA and <20% defluorination (as demonstrated by the concentration of fluoride released) was observed within 10 h (Fig. 1). There was no significant enhancement for the decomposition of PFOA when IAA, HDTMA-montmorillonite or IAA/Na^+^-montmorillonite was present (Fig. 1). In the reaction system containing IAA and HDTMA-montmorillonite, rapid degradation of PFOA was achieved. Nearly 100% of PFOA was degraded within 5 h (Fig. 1a). Additionally, we achieved over 90% defluorination within 10 h, which increased further to 96% after 18 h (Fig. 1b). The degradation and defluorination efficiencies were in the similar range compared to previous studies^22^,^23^ in which hydrated electrons were also used to treat PFOA (see Supplementary Table S1). However, in our system, the degradation/defluorination processes were not affected by dissolved oxygen; no statistically significant difference (*p *> 0.05) in PFOA degradation/defluorination occurred under aerobic vs. anaerobic conditions (Fig. 1). The degradation and defluorination of PFOS was essentially identical to those of PFOA suggesting the broad applicability of this novel chemistry to the degradation of PFCs (see Supplementary Fig. S1). Furthermore, the degradation/defluorination efficiency of PFOA was significantly enhanced by using a 500 W high pressure mercury lamp as the light source (emission spectrum shown in Supplementary Fig. S2b). For instance, PFOA was fully degraded within 90 min using 500 W mercury lamp vs. 5 h using 36 W lamp (Fig. 1a and see Supplementary Fig. S3a).

**Figure 2:**
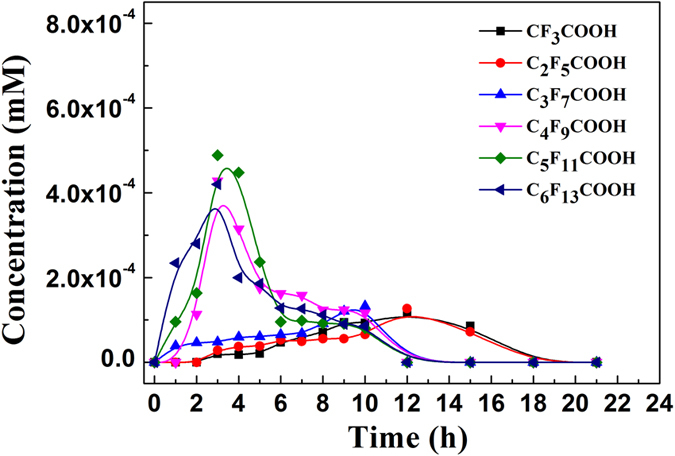
The production of short-chain perfluorinated acids as a function of irradiation time during the degradation of PFOA. Experimental conditions: the initial concentrations of PFOA, 3-indole-acetic-acid, and HDTMA-montmorillonite were 10 mg L^−1^, 1 mM and 2.2 g L^−1^, respectively; pH was adjusted to 6.0 by adding NaOH and HCl; a 36 W low-pressure mercury lamp was used to provide light irradiation.

The photoreduction pathway for PFOA consists of sequential defluorination accompanied by the simultaneous cleavage of the associated C-C bond. First of all, hydrated electrons can act as a nucleophile, leading to the reductive cleavage of C-F bonds, simultaneously, UV radiation of PFOA results in the cleavage of C-C bonds^22^,^23^. This is supported by the identification of lower molecular weight fluorinated carboxylic acids as the reaction intermediates (Fig. 2), which was also observed in prior studies^22^,^23^. Based on our previous work^27^,^28^, the C-16 alkyl chains of HDTMA are viewed as forming a hydrophobic phase in clay interlayer regions, which provides an effective sorptive phase for PFCs, and promotes their contact with hydrated electrons formed by light irradiation of IAA. We hypothesize that the hydrocarbon phase created by the overlapping of C-16 chains in the gallery regions protects the hydrated electrons from reaction with atmospheric oxygen, which is known to occur otherwise^29^,^30^.

### Adsorption isotherm and mechanism

To further test this hypothesis, we measured sorption isotherms of PFOA and IAA by Na^+^- and HDTMA-montmorillonite. All the isotherms were well-described using the Langmuir isotherm equation (r^2^ ≥ 0.92). The sorption of both PFOA and IAA by HDTMA-montmorillonite was much greater than that by Na^+^-montmorillonite (Table 1 and see Supplementary Fig. S4). As shown in Table 1, the maximum sorption capacity of PFOA and IAA on HDTMA-montmorillonite are 277.3 and 70.0 mmol kg^−1^, compared to 8.2 and 4.7 mmol kg^−1^ on Na-montmorillonite. The HDTMA-derived hydrocarbon phase present in clay interlayers is known to be an effective sorbent for a wide variety of organic compounds via a phase partition process^31^,^32^. These results are consistent with our previous study^24^ demonstrating that the photoreduction by hydrated electron occurs predominantly in the interlayer regions of montmorillonite clay, and that the sorption of PFCs and IAA by montmorillonite is a prerequisite for the effective photoreduction of PFCs in the reaction system described here.

### Effect of solution pH

**Figure 3:**
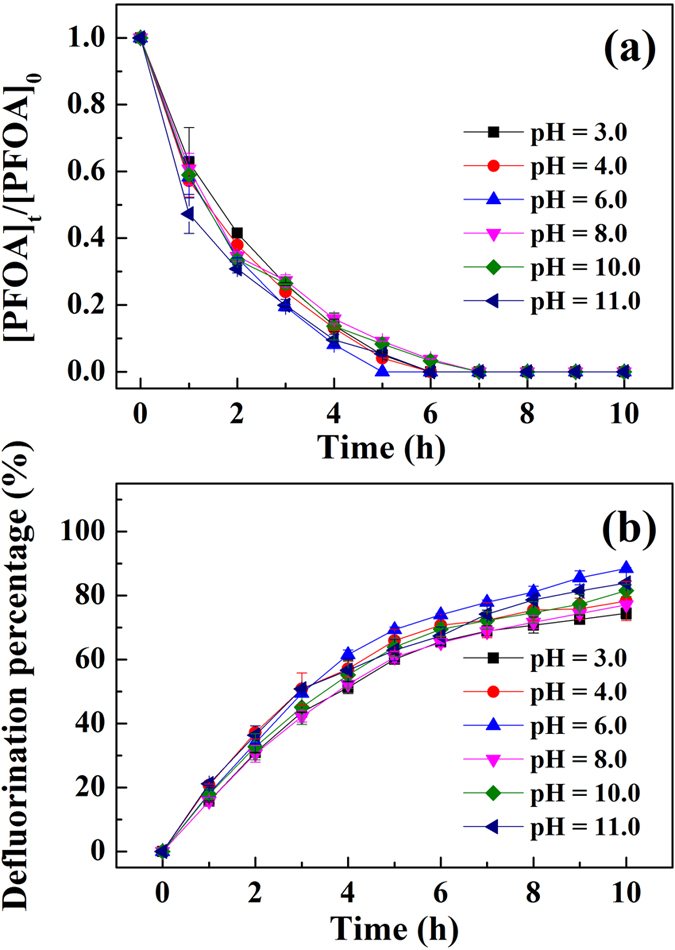
Effect of initial pHs on (**a**) photodegradation and (**b**) defluorination of PFOA by 3-indole-acetic-acid under the irradiation of a mercury lamp as a function of time in the presence of HDTMA-montmorillonite. Experimental conditions: the initial concentrations of PFOA, IAA, and clay mineral were 10 mg L^−1^, 1 mM and 2.2 g L^−1^, respectively; pH was adjusted to 3.0, 4.0, 6.0, 8.0, 10.0 and 11.0 by NaOH and HCl, respectively; a 36 W low-pressure mercury lamp was used to provide light irradiation.

The photodegradation experiments described above were performed at initial pH values ranging from 3.0 to 11.0. The degradation of PFOA and release of fluoride ion were almost not affected by pH (*p* > 0.05), only slight decrease at low pH was observed (Fig. 3). This contrasts to previous studies^23^ in which low pH significantly inhibited the degradation of PFOA. Even at pH 3.0, PFOA could be effectively degraded in our reaction systems, PFOA was fully decomposed in 7 h and the defluorination could reach 75% within 10 h. Whereas prior research by others showed that PFOA could not be reduced by hydrated electrons efficiently at pH < 8.0^23^. The unique structure of interlayer hydrophobic phase in HDTMA-montmorillonite apparently prevents the quenching of photo-generated hydrated electrons by protons. Our prior studies have clearly established that naturally occurring inorganic cations as well as protons in montmorillonite interlayers are effectively replaced by large organic cations, such as HDTMA^33^,^34^. Simultaneously, the interlayer water content is greatly reduced since the amount of interlayer water is dependent largely on the hydration energy of cations occupying the exchangeable sites of the clay^35^. Hence, organo-modification changes the micro-environment in clay interlayers in a manner that could plausibly prolong the lifetime of hydrated electrons. Furthermore, Xu and Boyd showed that HDTMA phase of such organo-clays provides hydrophobic barriers to the movement of small ions (e.g. protons) into the interlayer region^33^,^34^.

### EPR and FTIR analysis

**Figure 4:**
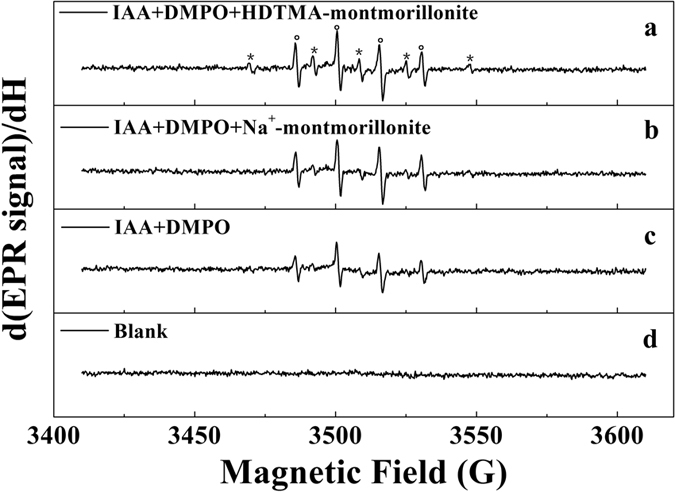
The first derivative EPR spectra of 3-indole-acetic-acid solution during continuous UV light irradiation in the presence of (**a**) HDTMA-montmorillonite and DMPO, (**b**) Na^+^-montmorillonite and DMPO, (**c**) DMPO, (**d**) HDTMA-montmorillonite. The DMPO-H and DMPO-OH lines are indicated by asterisks and circles, respectively. Experimental conditions: the initial concentrations of DMPO, IAA and clay mineral were 20 mM, 1 mM and 2.2 g L^−1^, respectively; pH was adjusted to 4.0 by adding NaOH and HCl; an arc light source equipped with a 350 W mercury lamp was used as the light source.

In this study, *in situ* EPR and FTIR spectroscopic analysis was conducted to investigate the reaction mechanism for degradation of PFCs by hydrated electrons. DMPO was used to scavenge the hydrated electrons and hydroxyl radicals formed in the reaction. The protonated DMPO-electron (DMPO-H) and the DMPO-OH spin adducts were detected during irradiation of a typical reaction mixture containing IAA, DMPO and HTDMA-montmorillonite (Fig. 4a). The spin Hamiltonian parameters observed for DMPO-H (a(N,NO) = 16.51 ± 0.01 G, a(H,CH2) = 22.59 ± 0.01 G) and DMPO-OH (a(N,NO) = 14.93 ± 0.01 G, a(H,C(OH)H) = 14.87 ± 0.01 G) spin adducts are in good agreement with the values reported in the literature^36^. For comparison, only the DMPO-OH spin adduct was detected in samples containing IAA or IAA/Na^+^-montmorillonite (Fig. 4b,c). The EPR spectral results strongly indicate that the presence of HDTMA-clay effectively inhibited the reaction between oxygen/proton and hydrated electrons, which is consistent with our observation that HDTMA-montmorillonite provides a reaction module for the effective degradation of PFCs by hydrated electrons generated from IAA.

**Figure 5:**
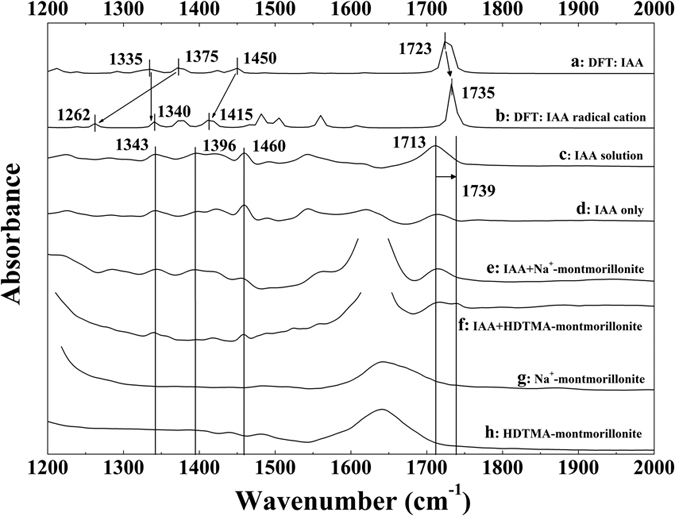
Comparison of calculated and experimental IR spectra: calculated IR spectra of (**a**) 3-indole-acetic-acid and (**b**) 3-indole-acetic-acid radical cation; observed IR spectra of (**c**) 3-indole-acetic-acid in solution, (**d**) 3-indole-acetic-acid solution irradiated by mercury lamp, and 3-indole-acetic-acid solution irradiated by mercury lamp in the presence of (**e**) Na^+^-montmorillonite, (**f**) HDTMA-montmorillonite; observed IR spectra of (**g**) Na^+^-montmorillonite, (**h**) HDTMA-montmorillonite irradiated by mercury lamp. Experimental conditions: the initial concentrations of 3-indole-acetic-acid, and clay mineral were 10 mM and 5 g L^−1^, respectively; pH was adjusted to 4.0 by adding NaOH and HCl; an arc light source equipped with a 350 W mercury lamp was used as the light source.

The major IR peaks of IAA were assigned (see Supplementary Table S2) in accordance with IR band assignments in the previous studies^37^. Irradiation of the IAA/HDTMA-montmorillonite system resulted in the emergence of a new peak at 1739 cm^−1^ (Fig. 5f). This peak appeared only during irradiation, and disappeared rapidly as light was off (see Supplementary Fig. S5). In experimental systems lacking IAA and/or HDTMA-montmorillonite, no such peak was observed (Fig. 5d,e,g,h and see Supplementary Figs S6–S9). Hence, this new light-induced peak was attributed to the formed IAA radical cations^24^,^38^.

**Figure 6:**
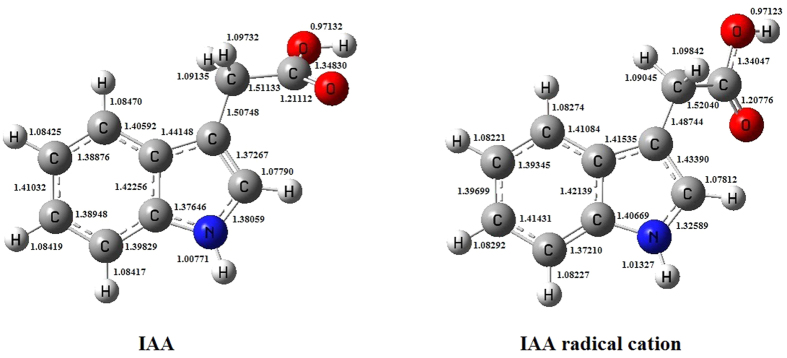
The optimized molecular structures of IAA and IAA radical cation as obtained from DFT calculations.

The IR spectral peak changes occurring during the irradiation of the clay/IAA suspension were evaluated by optimizing the geometries (Fig. 6) and calculating the theoretical spectra of gas phase IAA and the corresponding IAA radical cation (Fig. 5a,b). The computed spectrum of IAA was consistent with the observation from the experiment (Fig. 5a,c and see Supplementary Table S2). According to the calculated IR spectrum, 1375 and 1450 cm^−1^ peaks in the spectrum of molecular (neutral) IAA shift to 1262 and 1415 cm^−1^ upon formation of the IAA radical cation. The significant IR shifts of peaks at 1375 and 1450 cm^−1^ could be tentatively attributed to the substantial disturbance of the CH_2_ group caused by the extraction of one electron from the pyrrole ring. However, due to the strong IR bands of water and clay, only the highest intensity peak at 1739 cm^−1^ was observable for IAA radical cation in our experiments, which corresponds to C = O stretching. It shifts from 1713 to 1739 cm^−1^, in good agreement with the calculated shift from 1723 to 1735 cm^−1^ (see Supplementary Table S2).

The complexity of IAA/clay system might explain the slight discrepancy between experimental and computational spectra^38^. Hence, both FTIR and DFT theoretical calculation results in this study show strong evidence for the formation of the IAA radical cation in the system provided by HDTMA-montmorillonite. These results further demonstrate that the observed degradation of PFOA was caused by the hydrated electrons derived from IAA exposed to UV light irradiation. Importantly, the IAA radical cations produced after charge separation were stabilized in a micro-environment formed by HDTMA-montmorillonite, specifically the hydrophobic domains in clay interlayers prevent the formed hydrated electrons from reacting with oxygen/protons.

## Conclusions

Our experimental results clearly demonstrate that the degradation mechanism of PFCs by IAA catalyzed by HDTMA-montmorillonite can be envisioned as follows: IAA and PFCs molecules are initially adsorbed onto the surface of HDTMA-montmorillonite. With UV light irradiation, IAA molecules are photoexcited to produce hydrated electrons and IAA radical cations in clay interlayers. The unique structure of HDTMA-montmorillonite stabilizes the radical cations and prevents their recombination with hydrated electrons. Furthermore, the hydrophobic organic phase of HDTMA-montmorillonite acted to concentrate PFCs and IAA in the interlayer regions, thereby increasing the probability of contact between PFCs and the hydrated electrons generated from co-sorbed IAA, leading to the reductive defluorination of PFCs. Hence HDTMA-montmorillonite can be conceptualized as a unique nano-catalyst that localizes PFCs and IAA in a constrained space, stabilizes IAA radical cations hence promoting formation of hydrated electrons, and provides a matrix that shields the hydrated electrons from quenching by protons and oxygen.

In the HDTMA-montmorillonite/IAA system, PFCs can be efficiently defluorinated by photoradiation under mild conditions. Since both IAA and montmorillonite are naturally occurring substances, and HDTMA-montmorillonite has strong affinity for many persistent organic contaminants^39^,^40^, this unique “green chemistry” offers great potential to provide a cost-effective and environmental-friendly strategy for *in situ* or *ex situ* environmental remediation of waters and soils/sediments. Remarkably, these troublesome highly recalcitrant PFCs can be fully defluorinated and degraded using innocuous naturally occurring reagents, mild reaction conditions and low energy inputs.

## Materials and Methods

### Chemicals

Analytical grade IAA, sodium acetate, sodium chloride, sodium carbonate, sodium bicarbonate, acetic acid, 5,5-dimethy-1-pyrroline *N*-oxide (DMPO), hexadecyltrimethylammonium (HDTMA) bromide, PFOA and PFOS were all purchased from Sigma-Aldrich and the purities of the chemicals were >98%. Hydrochloric acid (HCl) and sodium hydroxide (NaOH) were obtained from Nanjing Chemical Regent CO., LTD (Nanjing, China). HPLC grade acetonitrile and methanol were from Merck (Darmstadt, Germany). All the chemicals were used without any further purification.

### Preparation of homoionic Na^+^- and HDTMA-montmorillonite

Na^+^ saturated montmorillonite supplied from Fenghong Chemical Co. (Zhejiang, China) was prepared following the method of Arroyo *et al*.^41^. Initially, carbonate impurities in montmorillonite were removed by titrating the clay suspension to pH 6.8 using 0.5 M sodium acetate buffer (pH = 5.0), then the clay suspension was centrifuged at 60 g for 6 min to obtain clay sized particles (<2 μm). The clay fraction was resuspended in 0.1 M NaCl solution and centrifuged at 3500 g for 15 min. To ensure full saturation of montmorillonite by Na^+^, this procedure was repeated five more times. The resultant Na^+^-montmorillonite was washed by deionized water until a negative chloride test using AgNO_3_, finally freeze-dried for further use. The HDTMA-montmorillonite was prepared according to the method described by Boyd *et al*.^27^. Specifically, 50 g of the obtained Na^+^-montmorillonite was suspended in 500 mL water for 8 h. Aqueous solution containing 38.5 mmol HDTMA bromide was added into the Na^+^-montmorillonite suspension and the amount of HDTMA was equal to the CEC sites of the clay in suspension. The HDTMA-montmorillonite was then washed and freeze-dried following the procedure described above.

### Batch sorption experiments of IAA and PFOA on Na^+^- and HDTMA-montmorillonite

A batch equilibrium method was used to investigate the sorption of IAA and PFOA on Na^+^- and HDTMA-montmorillonite. For each isotherm experiment, a 15 mL glass centrifuge tube received a weighed amount of Na^+^- or HDTMA-montmorillonite clay (22 mg) in 10.0 mL of PFOA or IAA solution. The initial concentration range for PFOA was from 0.00483 to 2.415 mM, and from 0.05 to 2.1 mM for IAA. The pH was controlled at 6.0 ± 0.1 by adding NaOH or HCl. To avoid the exposure to light, the centrifuge tubes were covered with aluminum foil and shaken at 50 rpm on a rotary shaker at room temperature for 24 h, and then centrifuged at 1500 g for 15 min. The preliminary experiments demonstrated that during the sorption process, the loss of sorbates due to other transformation was negligible and sorption could reach equilibrium within 24 h. The concentration of PFOA in supernatant was determined by a HPLC system (Waters Alliance 2695, Milford, MA) with a 4.6 × 250 mm Waters X-Bridge Shield C18 column followed by a Waters conductivity detector. The mobile phase consisted of 40% acetonitrile and 60% 0.02 M ammonium acetate at a flow rate of 1 mL min^−1^. The concentration of IAA was analyzed by the HPLC with the same column as mentioned above. A Waters 2998 photodiode array detector (at 262 nm) was utilized and the mobile phase was a mixture of 35% acetonitrile and 65% water containing 5‰ acetic acid with the flow rate of 1 mL min^−1^. The amount of sorption on clay mineral surface was calculated by subtracting the mass in supernatant phase from the mass in blank sample (no clay).

### Degradation of perfluorinated compounds

The decomposition of PFOA by hydrated electrons was conducted in a photochemical reactor (model XPA-7, Xujiang Electromechanical Inc., Nanjing, China) equipped with a cylindrical quartz photoreactor (height = 400 mm; internal diameter = 40 mm). The initial concentration of PFOA, IAA and clay mineral was 10 mg L^−1^, 1 mM and 2.2 g L^−1^, respectively. Photodegradation experiment was performed by internal irradiation with a low-pressure mercury lamp (Philips; 254 nm, 36 W) immerged into the reaction solution. A radiometer (CEL-NP2000-10, Ceaulight Inc., Beijing, China) was used to measure the light intensity on the surface of the lamp sheath as 4.5 mW cm^−2^. The reaction temperature was maintained at 25 °C through a water bath. To study the pH effect on the degradation process, the initial pH of the suspension was adjusted to 3.0, 4.0, 6.0, 8.0, 10.0 and 11.0 by adding 0.1 M HCl or NaOH. Similar experiment was also conducted under anaerobic condition by continuously purging the system with N_2_, the dissolved oxygen content in the system decreased from 7.16 to 0.2 mg L^−1^ after deoxygenation. During the kinetic experiment, 5 mL of reaction suspension was withdrawn at predetermined time intervals to determine the concentrations of PFOA and released fluoride ion.

For PFOA measurement, 1 mL sample was transferred to 8 mL glass centrifuge tube and 2 mL acetonitrile was added to extract the adsorbed PFOA for 10 h, then centrifuged at 1500 g for 15 min. Our preliminary experiment showed that the recovery of extraction for PFOA was >98%. The concentration of PFOA in supernatant was analyzed by the developed method as described above. Fluoride ions in the samples were quantified with the ion chromatography (ICS-900, DIONEX Co., USA) equipped with an anion exchange column (Dionex IonPac AS23, 4 mm i.d. × 250 mm) and an auto-sampler. The samples were filtered through 0.22 μm nylon filter membranes before the analysis. The mobile phase was a mixture of NaHCO_3_ (1.6 mM) and Na_2_CO_3_ (10 mM) and the flow rate was 1 mL min^−1^. The suppressor current was set at 51 mA. The concentrations of short-chain perfluorinated acids (C2~C7) formed during the reaction were determined by a Waters ACQUITY UPLC system (Waters, Milford, MA, USA) combined with negative ion electronspray tandem mass spectrometry. To reduce the background signal, a perfluorochemical isolator column was placed in-line between the solvent mixer and the injector. An ACQUITY BEH C18 column (2.1 mm × 50 mm, 1.7 μm, Waters, Milford, MA, USA) was used for separation and the column temperature was maintained at 50 °C. The mobile phase consisted of 2 mM ammonium acetate in water (A) and methanol (B) with a constant flow rate of 0.4 mL min^−1^. The following eluent gradient was applied: starting with 10% of B for 0.5 min, and increasing to 25% of B until 1 min, then linear gradient to 85% of B until 6 min, after keeping 100% of B until 7 min, finally returning to the starting condition within 1 min and holding for another 1 min for equilibration. The photodefluorination of PFOS under the similar reaction conditions was also studied. All degradation experiments were conducted in triplicate.

### *In situ* Fourier transform infrared and electron paramagnetic resonance measurements

*In situ* electron paramagnetic resonance (EPR) measurements were carried out on a Bruker EMXplus EPR spectrometer at room temperature. The instrumental parameters for EPR analysis were: microwave frequency, 9.85 GHz; modulation frequency, 100 kHz; microwave power, 20 mW; modulation amplitude, 1 G; receiver gain, 1.0 × 10^3^; sweep width, 200 G; center field, 3510 G. For the EPR measurement, 20 μL sample containing 1 mM IAA, 20 mM DMPO (radical trap agent to scavenge the generated radicals) and 2.2 g L^−1^ montmorillonite clay was added into a quartz capillary EPR tube with the internal diameter of 1.0 mm (707-SQ-250M, Wilmad, USA). An arc light source equipped with a high pressure 350 W mercury lamp (CEL-350, Ceaulight, Beijing) was used to provide *in situ* irradiation. The photoreaction was initiated by the UV light passing through the window-shades of the resonant cavity into the sample. The irradiation spectrum of the high pressure mercury lamp used in this study is shown in Supplementary Fig. S2a.

A Fourier transform infrared (FTIR) spectrometer (Bruker tensor 27, Ettlingen, Germany) was employed for *in situ* FTIR analysis. The FTIR data were collected by a liquid nitrogen cooled mercury cadmium telluride detector with total of 64 scans and a resolution of 4 cm^−1^. A 16 bounds zinc selenide attenuated total reflection (ATR) liquid cell accessory (Pike Technology, Madison, WI) was used. For sample preparation, 50 mg clay was initially mixed with 10 mL IAA solution (10 mM) and shaken on a rotary shaker in the dark at 50 rpm for 2 h. After the suspension mixture was centrifuged at 1500 g for 15 min, the supernatant was discarded and the residue clay paste was spread onto the ATR crystal surface. The UV irradiation was produced by a 350 W mercury lamp (same as described above in EPR measurement), which was placed ~25 cm above the ATR cell. This setting allows us to *in situ* monitor the spectral changes upon irradiation. The background IR spectrum of clay paste in the absence of IAA was also collected and the spectrum of IAA solution (10 mM) only was used for comparison. The IR spectrum corresponding to IAA in IAA/clay mixture was obtained by subtracting the background signal (clay paste spectrum) from the observed spectrum of IAA/clay paste.

### Quantum chemical calculations

The infrared spectra and molecular structure of IAA and IAA radical cation were calculated based on density functional theory (DFT) using B3LYP methods^42^ in Gaussian03 suit quantum chemical calculation programs^43^. The 6–311 ++ g(d,p) basis set was applied for all the atoms in IAA and IAA radical cation, including C, H, O and N. Gview program was used to construct the molecular structures based on the optimized calculation results.

## Additional Information

**How to cite this article**: Tian, H. *et al*. Complete Defluorination of Perfluorinated Compounds by Hydrated Electrons Generated from 3-Indole-acetic-acid in Organomodified Montmorillonite. *Sci. Rep*. **6**, 32949; doi: 10.1038/srep32949 (2016).

## Supplementary Material

Supplementary Information

## Figures and Tables

**Table 1:** Langmuir isotherm parameters for PFOA and 3-indole-acetic-acid adsorption on Na^+^-, and HDTMA-montmorillonite at pH 6.0.

Smectite clay	PFOA			IAA		
	*K*_L_ (L mmol^−1^)	*C*_max_ (mmol kg^−1^)	R^2^	*K*_L_ (L mmol^−1^)	*C*_max_ (mmol kg^−1^)	R^2^
Na^+^-montmorillonite	0.539	8.199	0.987	4.025	4.673	0.899
HDTMA-montmorillonite	11.839	277.312	0.944	8.212	70.005	0.929

The adsorption data were fitted with *Langmuir* isotherm model: *q*_e_ = (*K*_L_ × *C*_max_ × *C*_e_)/(1 + *K*_L_ × *C*_e_); where *q*_e_ is the amount of adsorbate adsorbed on mineral surfaces in mmol kg^−1^, *C*_e_ is the equilibrium concentration in mM, *K*_L_ is the Langmuir constant, L mmol^−1^; *C*_max_ is sorption capacity in mmol kg^−1^; R^2^ is the coefficient of determination from the overall regression analysis.
