# Supplementary material for: Complete Defluorination of Perfluorinated Compounds by Hydrated Electrons Generated from 3-Indole-acetic-acid in Organomodified Montmorillonite
Source: Sci Rep. 2016 Sep 9;6:32949. doi: 10.1038/srep32949 (PMC5017190; doi:10.1038/srep32949)
Supplement: Supplementary Information [file srep32949-s1.doc]

**Supplementary information for:**

**Complete Defluorination of Perfluorinated Compounds by Hydrated Electrons Generated from 3-Indole-acetic-acid in Organomodified Montmorillonite**

Haoting Tian^1^, Juan Gao^2^, Hui Li^3^, Stephen A. Boyd^3^, Cheng Gu^1^*

^1^State Key Laboratory of Pollution Control and Resource Reuse, School of the Environment, Nanjing University, Nanjing 210023, P.R. China

^2^Key Laboratory of Soil Environment and Pollution Remediation, Institute of Soil Science, Chinese Academy of Sciences, Nanjing, Jiangsu 210008, P. R. China

^3^Department of Plant, Soil and Microbial Sciences, Michigan State University, East Lansing, Michigan 48824, United States

*To whom correspondence should be addressed

Cheng Gu

Professor

School of the Environment, Nanjing University

Nanjing, Jiangsu, 210023, P. R. China

Phone/Fax: +86-25-89680636

E-mail: [chenggu@nju.edu.cn](mailto:chenggu@nju.edu.cn)

**Table S1.** Comparison of different treatments for PFOA degradation.

| **Technique** | **Conditions** | **Appropriate solution pH** | **Aerobic or anaerobic** | **Degradation efficiency** | **Defluorination efficiency** | **Ref.** |
| --- | --- | --- | --- | --- | --- | --- |
| Electrochemical oxidation | 0.114 mM of PFOA;  current density = 23.24 mA cm^−2^;  electrolyte = 10 mM NaClO_4_;  pH = 9.0;  reaction volume = 40 mL;  reaction time = 2 h | 3.0 - 12.0 | aerobic | ~100 % | 60 % | Zhuo et al., 2012 |
| Electrochemical oxidation | 0.241 mM of PFOA;  current density = 10 mA cm^−2^;  electrolyte = 10 mM NaClO_4_;  pH = 5.0;  reaction volume = 100 mL;  reaction time = 1.5 h | 3.0 - 9.0 | aerobic | 90 % | 73 % | Lin et al., 2012 |
| Sonochemical degradation | 0.0241 mM of PFOA;  *f* = 200 kHz;  pH = 4.7;  reaction volume = 60 mL;  reaction time = 1 h | 3.0 - 11.0 | anaerobic | 85 % | 70 % | Moriwaki et al., 2005 |
| Sonochemical degradation | 0.241 μM of PFOA;  *f* = 612 kHz;  pH = 4.7;  reaction volume = 600 mL;  reaction time = 2 h | 3.0 - 11.0 | anaerobic | ~100 % | 95 % | Cheng et al., 2010 |
| Direct photolysis | 0.0241 mM of PFOA;  λ = 253.7 nm;  15 W low-pressure mercury lamp;  pH = 9.0;  reaction volume = 740 mL;  reaction time = 14 h | 4.0 - 12.0 | anaerobic | ~25 % | 5 % | Qu et al., 2010 |
| Hydrated electron reduction (UV/KI) | 0.0241 mM of PFOA;  λ = 253.7 nm;  15 W low-pressure mercury lamp;  KI = 0.3 mM;  pH = 9.0;  reaction volume = 740 mL;  reaction time = 14 h | 8.0 - 12.0 | anaerobic | ~100 % | 98 % | Qu et al., 2010 |
| Hydrated electron reduction (UV/SO_3_^2-^) | 0.02 mM of PFOA;  λ = 253.7 nm;  10 W low-pressure mercury lamp;  SO_3_^2-^ = 10 mM;  pH = 9.0;  reaction volume = 200 mL;  reaction time = 24 h | 8.0 - 12.0 | anaerobic | 100 % | 85 % | Song et al., 2013 |
| Hydrated electron reduction (UV/IAA/ HDTMA-montmorillonite) | 0.0241 mM of PFOA;  λ = 253.7 nm;  36 W low-pressure mercury lamp;  IAA = 1.0 mM;  HDTMA-montmorillonite = 2.2 g L^-1^;  pH = 6.0;  reaction volume = 300 mL  reaction time = 10 h | 4.0 - 10.0 | aerobic | ~100 % | 90 % | Current study |

**Table S2.** Calculated and experimentally observed main FTIR frequencies of IAA and IAA radical cation in the region between 1200 and 2000 cm^-1^.

| IAA | | IAA radical cation | | assignments |
| --- | --- | --- | --- | --- |
| ν_exp_ (cm^-1^) | ν_cal_ (cm^-1^) | ν_exp_ (cm^-1^) | ν_cal_ (cm^-1^) |
| 1343 | 1335 |  | 1340 | benzene ring stretching and C-OH stretching |
| 1396 | 1375 |  | 1262 | CH_2_ wagging and synchronous bending of hydrogen in both rings |
| 1460 | 1450 |  | 1415 | CH_2_ scissoring |
| 1713 | 1723 | 1739 | 1735 | C=O stretching |


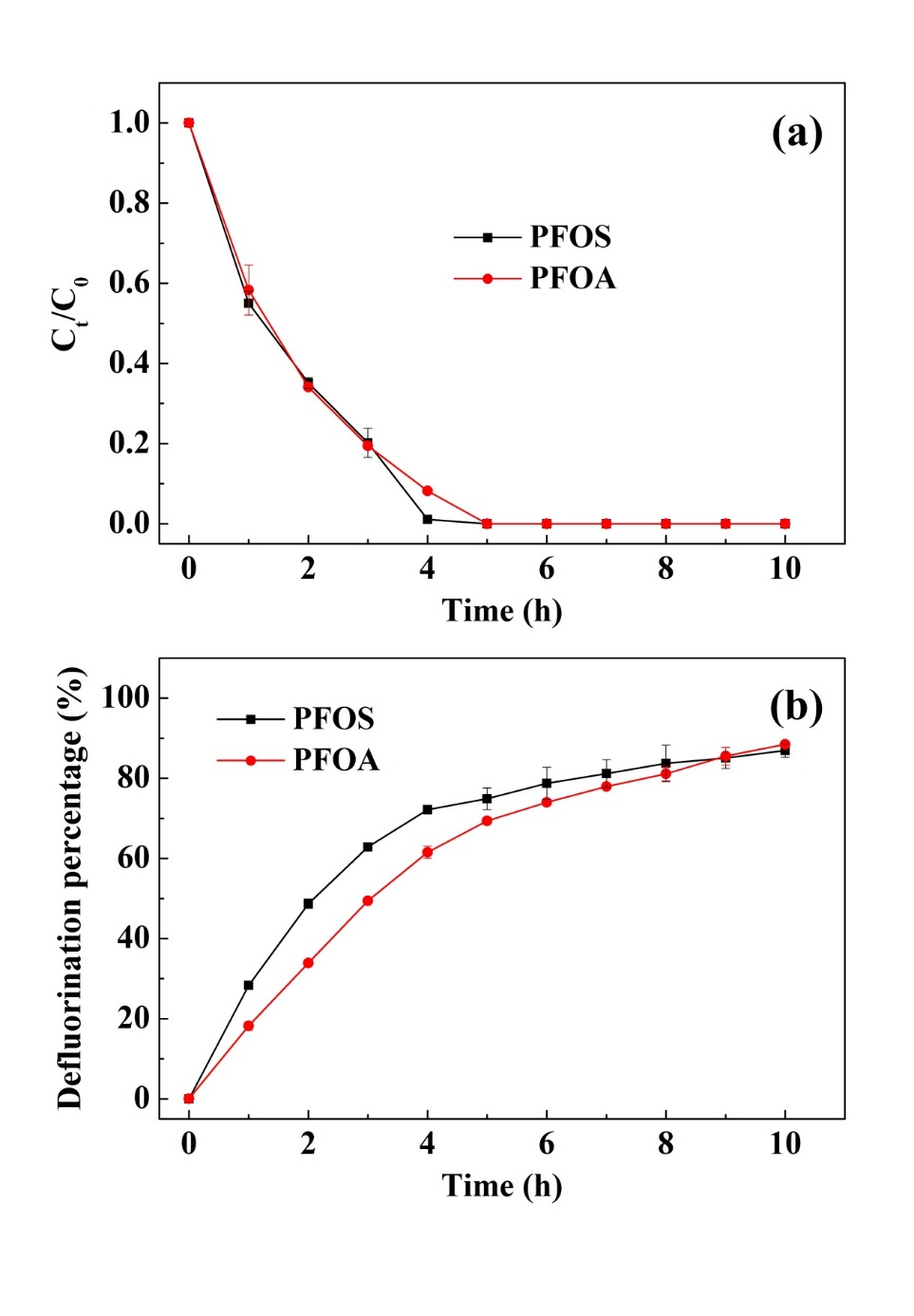


**Figure S1.** (**a**) Photodegradation and (**b**) defluorination of PFOS by 3-indole-acetic-acid under the irradiation of a mercury lamp as a function of time in the presence of HDTMA-montmorillonite. Experimental conditions: the initial concentrations of PFOS, 3-indole-acetic-acid, and clay mineral were 10 mg L^-1^, 1 mM and 2.2 g L^-1^, respectively; pH was adjusted to 6.0 by adding NaOH and HCl; a 36 W low-pressure mercury lamp was used to provide light irradiation.


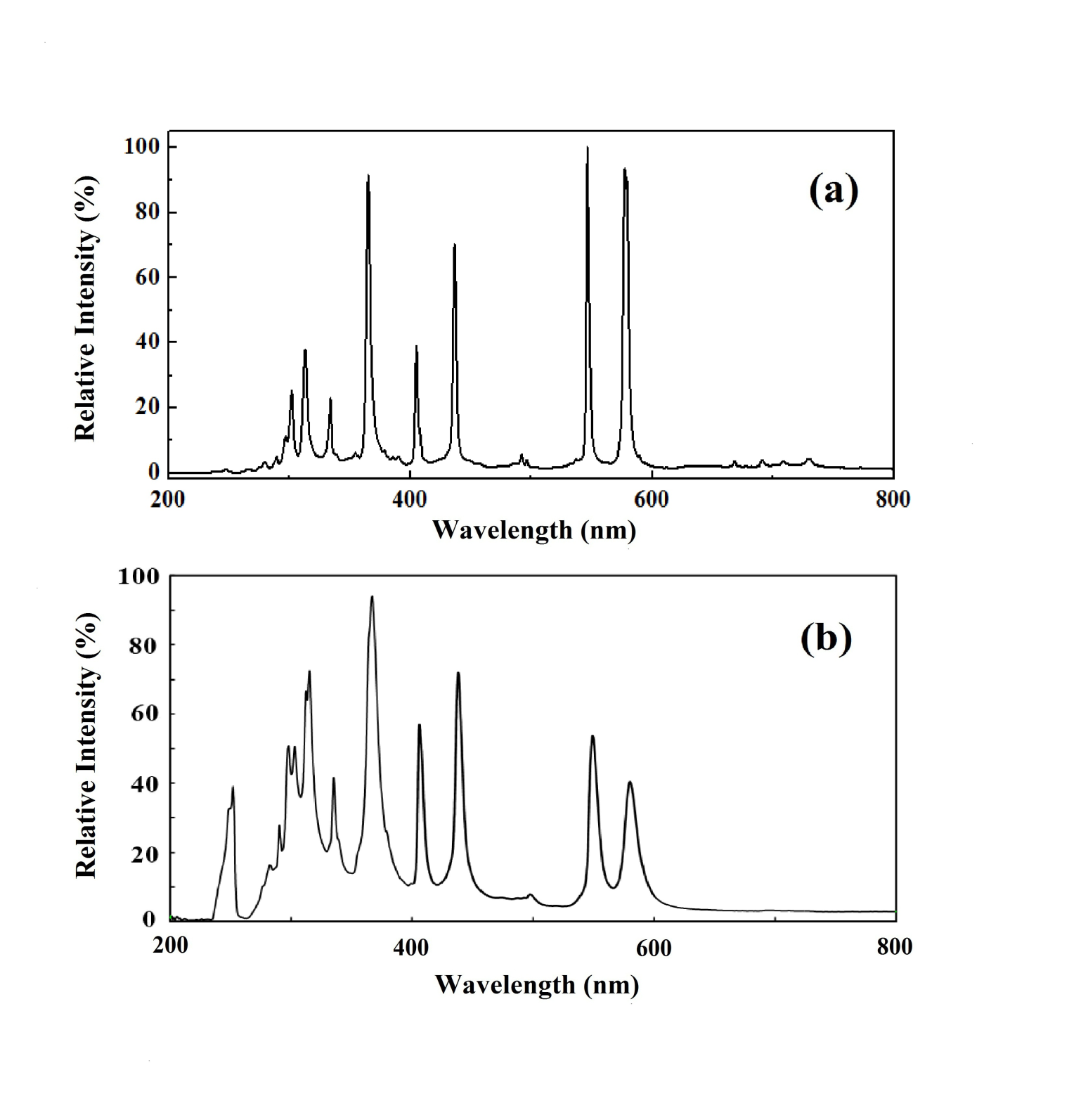


**Figure S2.** (a) Emission spectrum of the mercury lamp used in EPR and FTIR experiments, (b) Emission spectrum of the mercury lamp used in photodegradation experiments.


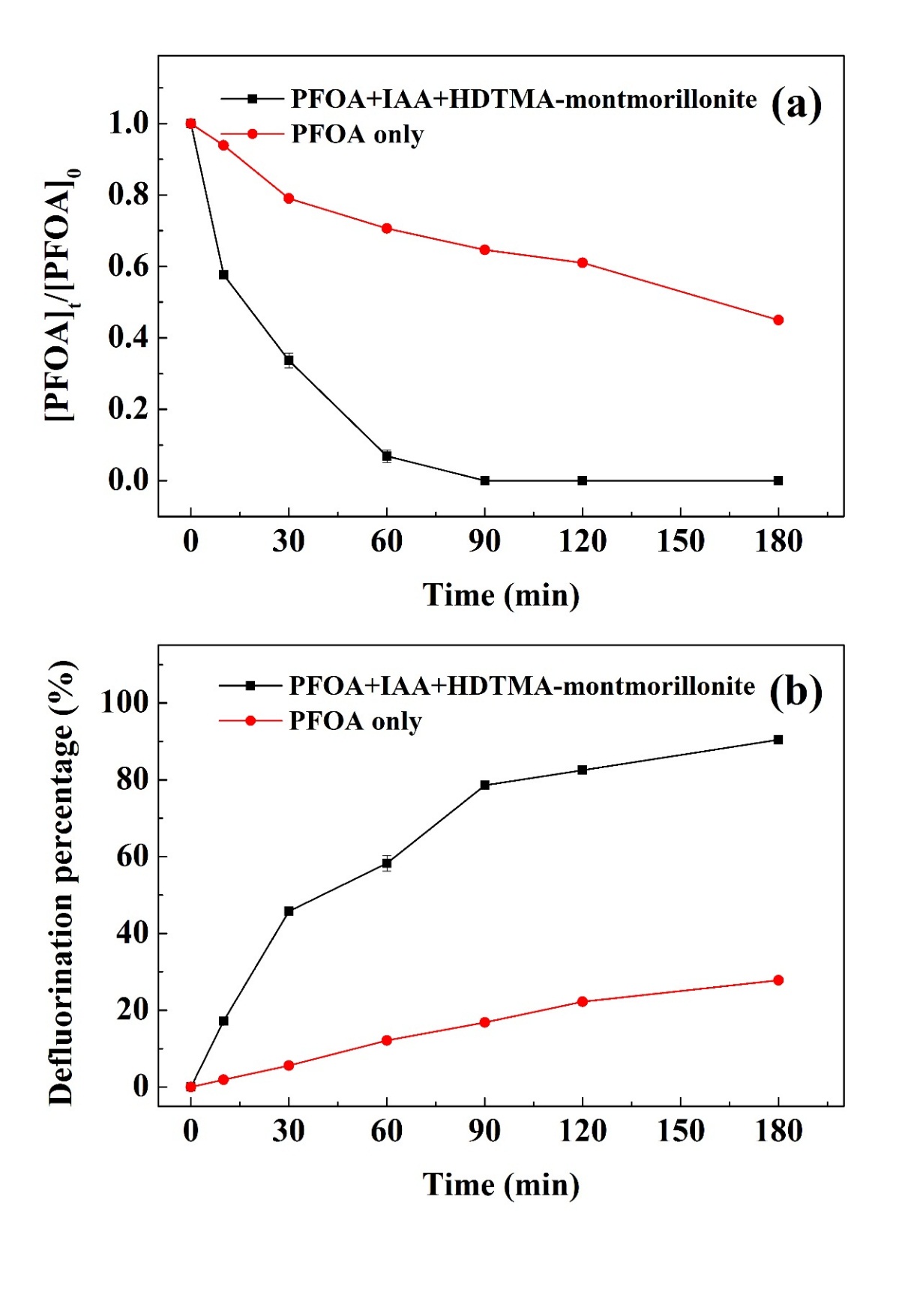


**Figure S3.** (**a**) Photodegradation and (**b**) defluorination of PFOA by 3-indole-acetic-acid under the irradiation of a mercury lamp as a function of time in the presence of HDTMA-montmorillonite. Experimental conditions: the initial concentrations of PFOA, 3-indole-acetic-acid, and clay mineral were 10 mg L^-1^, 1 mM and 2.2 g L^-1^, respectively; pH was adjusted to 6.0 by adding NaOH and HCl; a 500 W high-pressure mercury lamp was used to provide light irradiation. Error bars are the standard deviations of triplicate analyses.


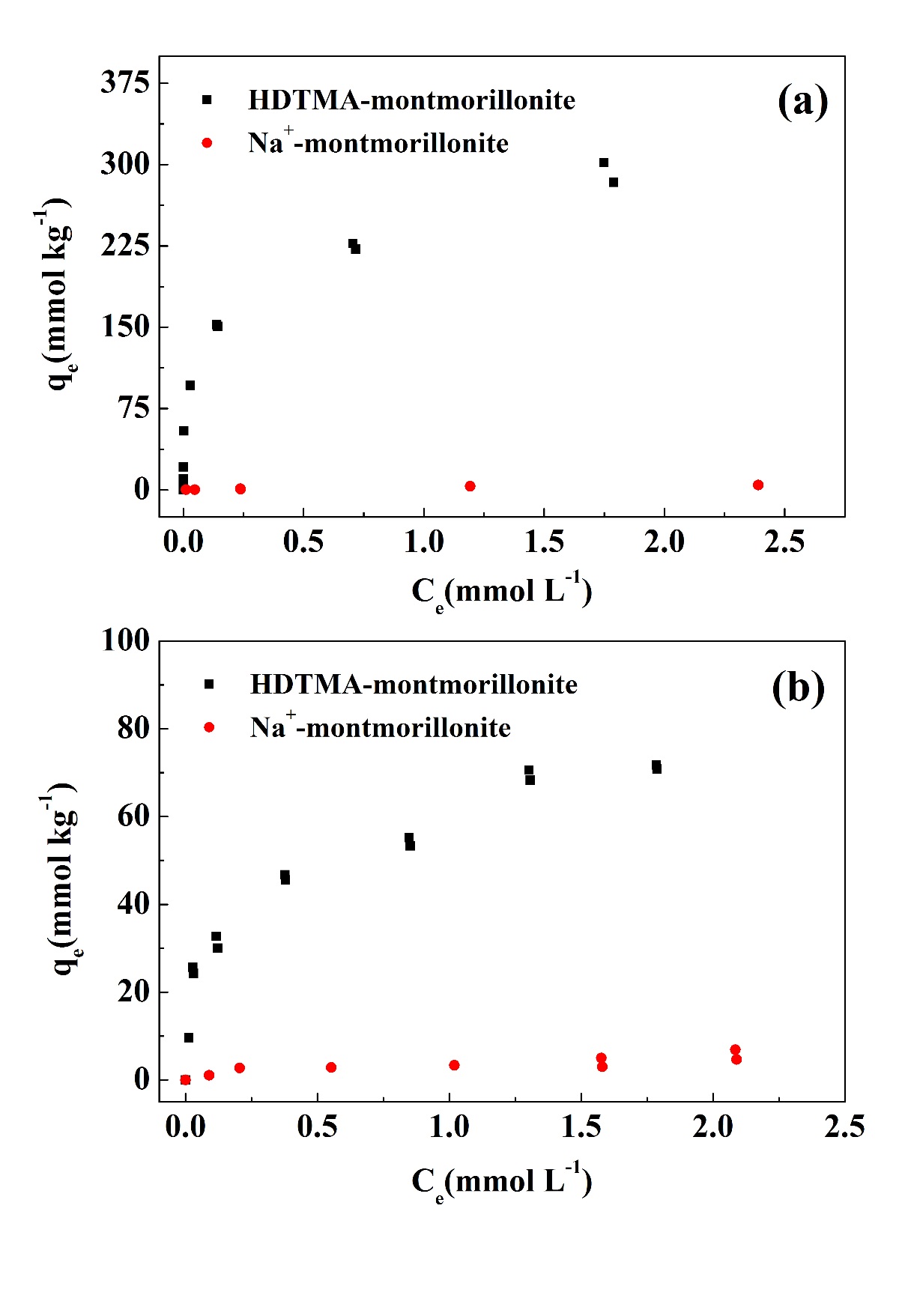


**Figure S4.** Sorption isotherms of (**a**) PFOA and (**b**) 3-indole-acetic-acid on Na^+^- and HDTMA-montmorillonite. *q*_e_ is the amount of PFOA (or 3-indole-acetic-acid) adsorbed on montmorillonite clay mineral in mmol kg^-1^, *C*_e_ is the equilibrium concentration of PFOA (or 3-indole-acetic-acid) in mM. Experimental conditions: 22 mg montmorillonite; PFOA with initial concentrations ranging from 0.00483 to 2.415 mM; 3-indole-acetic-acid with initial concentrations ranging from 0.05 to 2.1 mM; reaction medium: 10 mL aqueous solution (pH = 6.0, adjusted by adding NaOH and HCl).


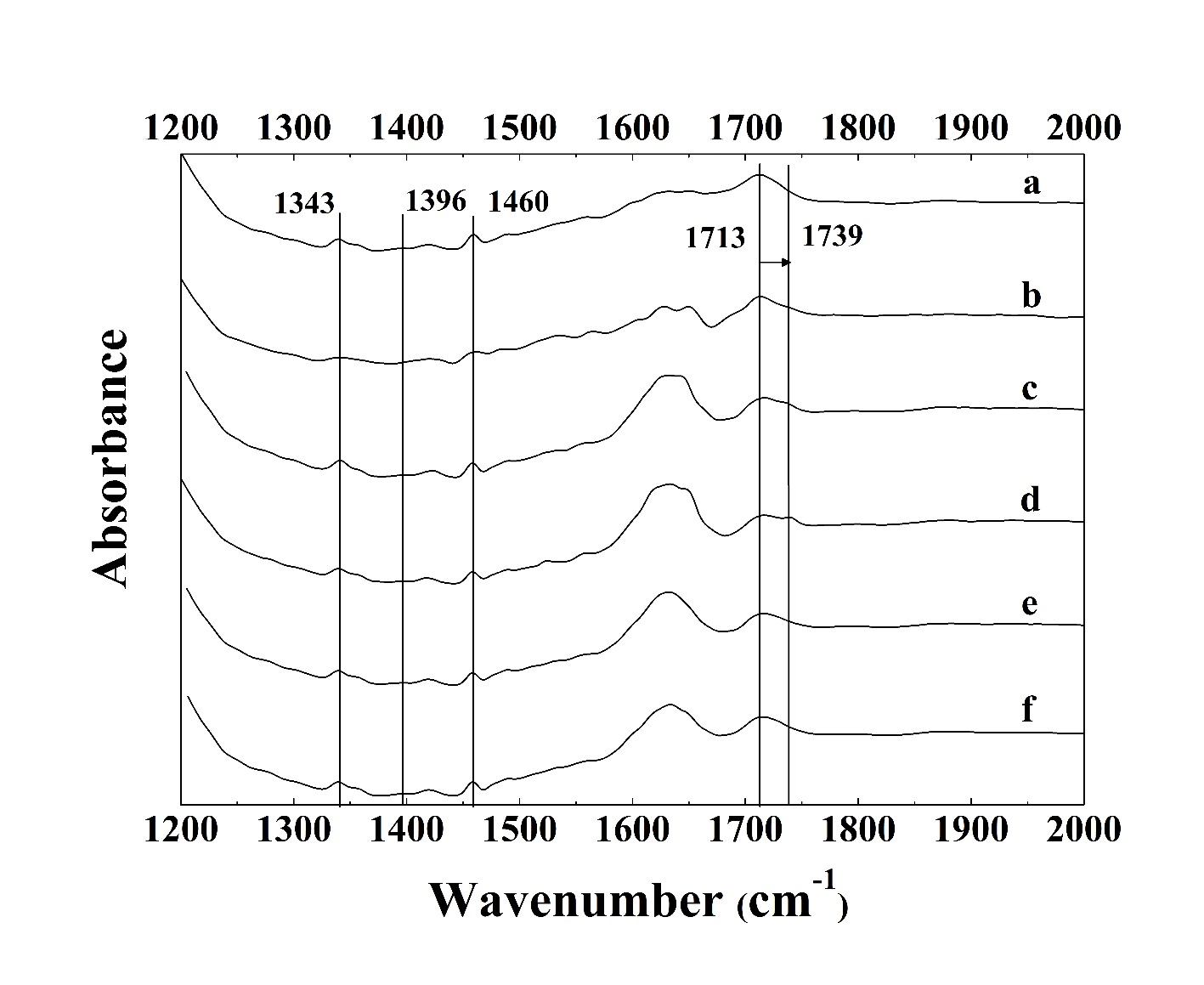


**Figure S5.** *In situ* FTIR spectra of IAA/HDTMA-montmorillonite mixture under mercury lamp as a function of irradiation time: a) 0, b) 1, c) 2, and d) 3 min. After obtaining spectrum at t = 3 min, the light was turned off, IR spectra were then collected at e) 1 and f) 3 min after light was off. Experimental conditions: the initial concentrations of 3-indole-acetic-acid, and clay mineral were 10 mM and 5 g L^-1^, respectively; pH was adjusted to 4.0 by adding NaOH and HCl; an arc light source equipped with a 350 W mercury lamp was used as the light source.


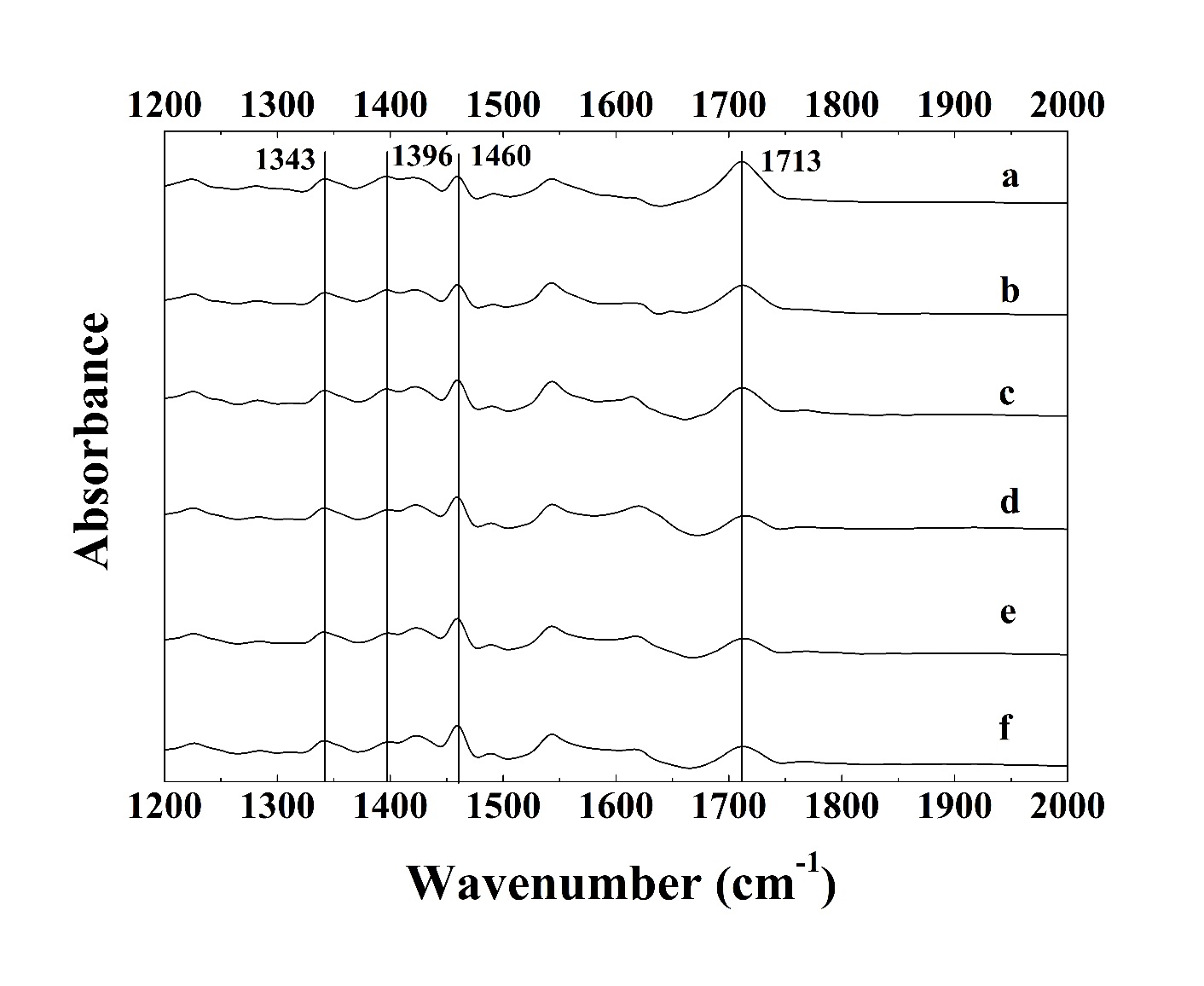


**Figure S6.** *In situ* FTIR spectra of 3-indole-acetic-acid solution under mercury lamp as a function of irradiation time: a) 0, b) 1, c) 2, and d) 3 min. After obtaining spectrum at t = 3 min, the light was turned off, IR spectra were then collected at e) 1 and f) 3 min after light was off. Experimental conditions: the initial concentration of 3-indole-acetic-acid was 10 mM; pH was adjusted to 4.0 by adding NaOH and HCl; an arc light source equipped with a 350 W mercury lamp was used as the light source.


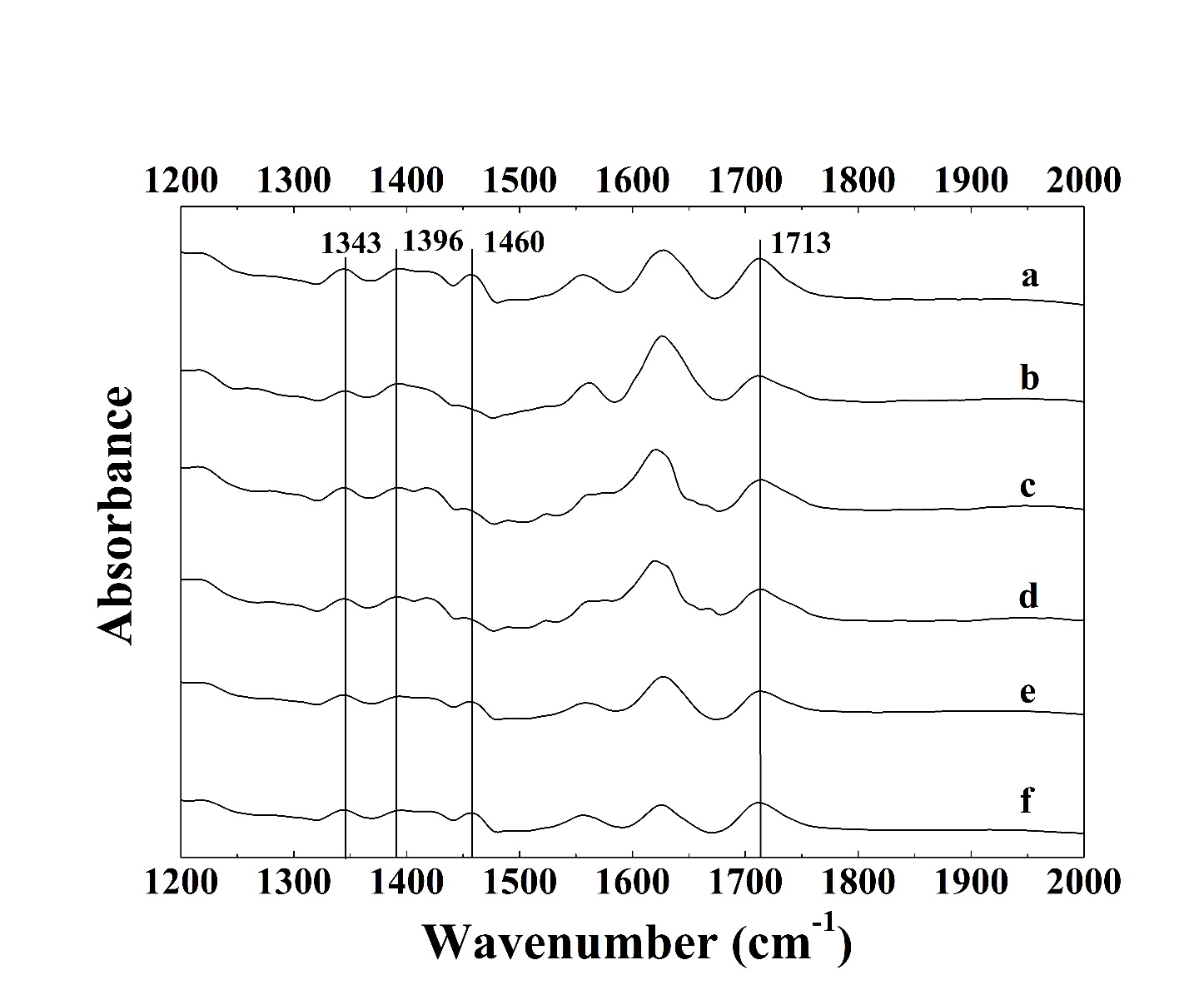


**Figure S7.** *In situ* FTIR spectra of IAA/Na^+^-montmorillonite mixture under mercury lamp as a function of irradiation time: a) 0, b) 1, c) 2, and d) 3 min. After obtaining spectrum at t = 3 min, the light was turned off, IR spectra were then collected at e) 1 and f) 3 min after light was off. Experimental conditions: the initial concentrations of 3-indole-acetic-acid, and clay mineral were 10 mM and 5 g L^-1^, respectively; pH was adjusted to 4.0 by adding NaOH and HCl; an arc light source equipped with a 350 W mercury lamp was used as the light source.


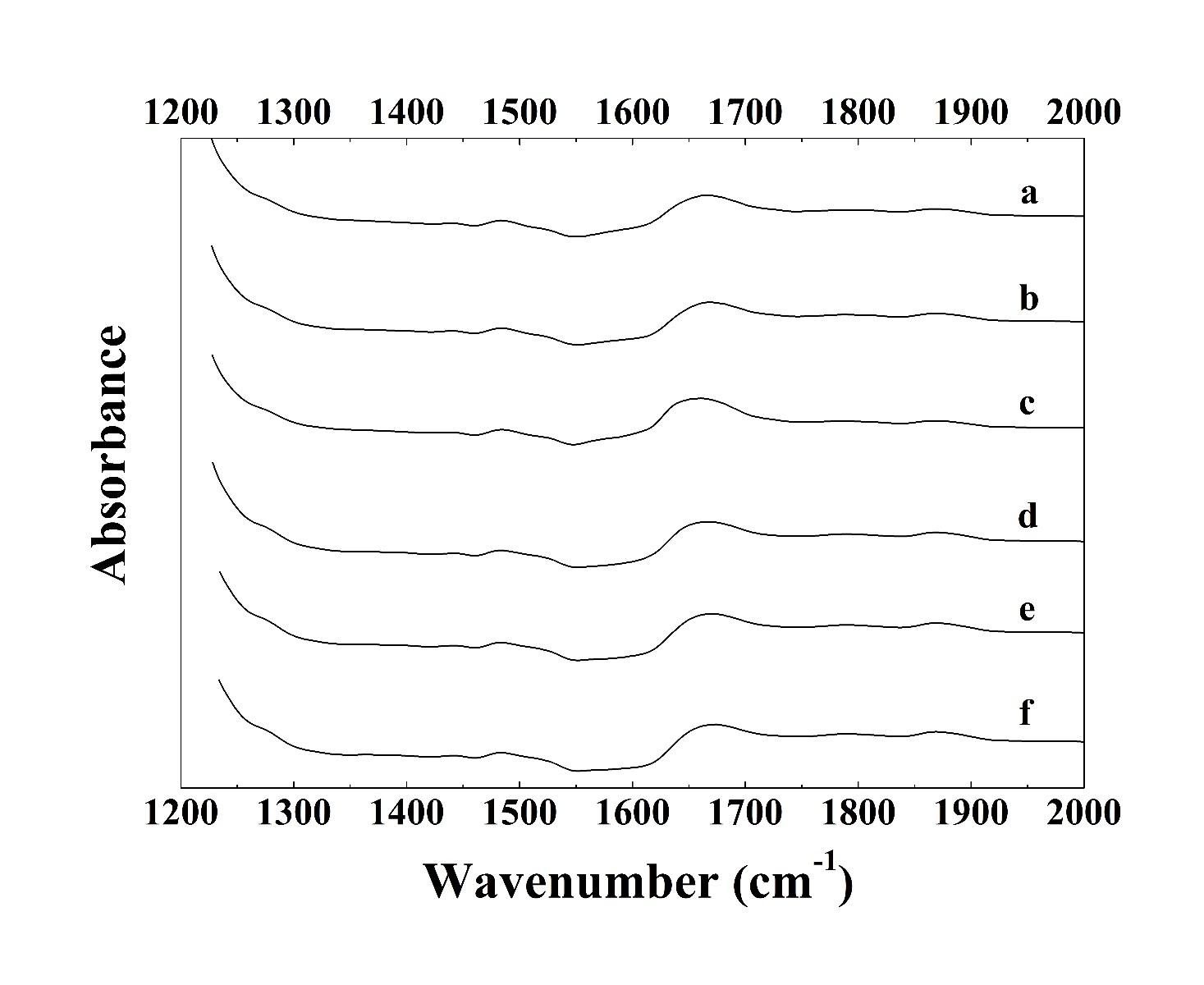


**Figure S8.** *In situ* FTIR spectra of Na^+^-montmorillonite paste under mercury lamp as a function of irradiation time: a) 0, b) 1, c) 2, and d) 3 min. After obtaining spectrum at t = 3 min, the light was turned off, IR spectra were then collected at e) 1 and f) 3 min after light was off. Experimental conditions: the initial concentration of clay mineral was 5 g L^-1^; pH was adjusted to 4.0 by adding NaOH and HCl; an arc light source equipped with a 350 W mercury lamp was used as the light source.


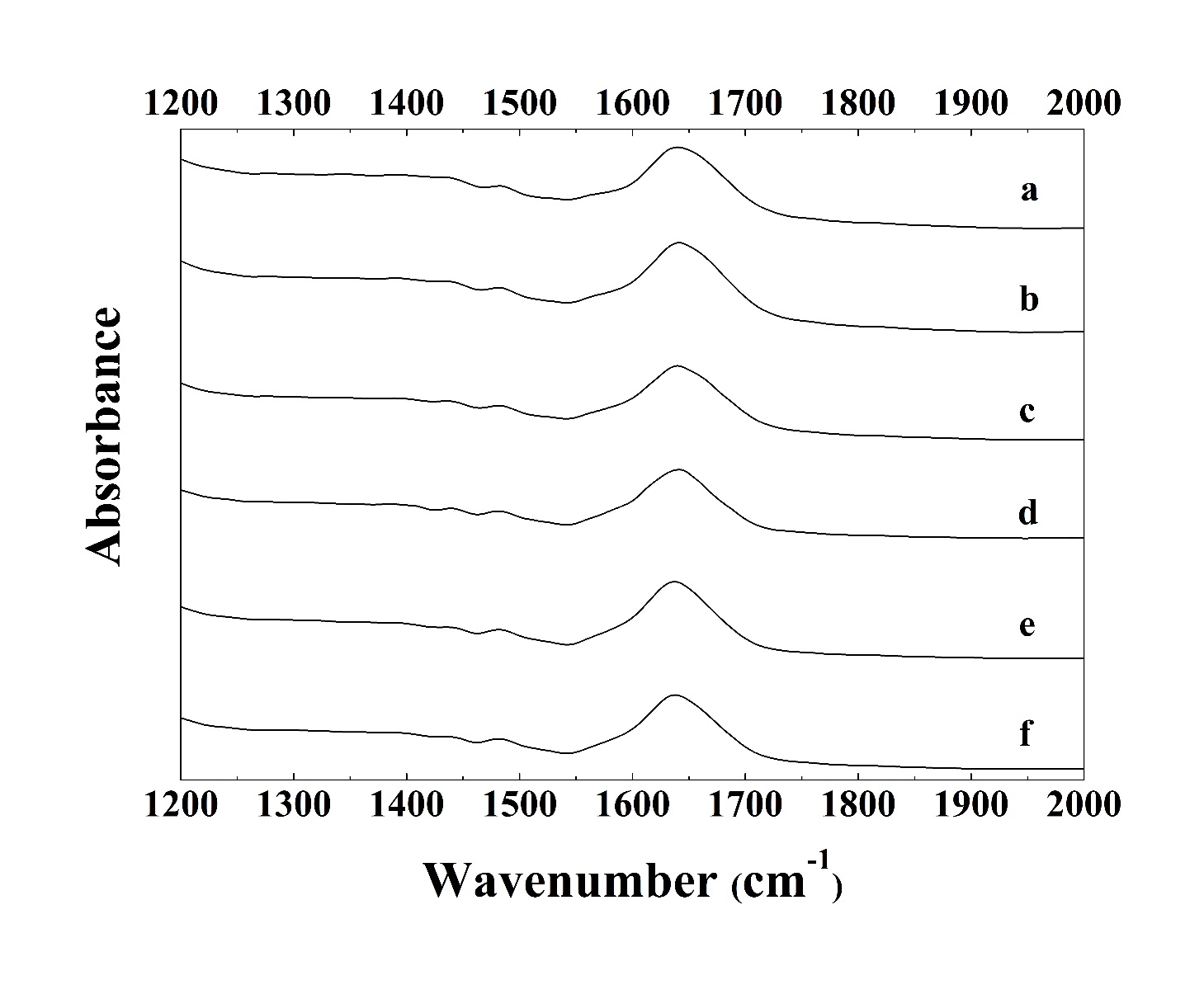


**Figure S9.** *In situ* FTIR spectra of HDTMA-montmorillonite paste under mercury lamp as a function of irradiation time: a) 0, b) 1, c) 2, and d) 3 min. After obtaining spectrum at t = 3 min, the light was turned off, IR spectra were then collected at e) 1 and f) 3 min after light was off. Experimental conditions: the initial concentration of clay mineral was 5 g L^-1^; pH was adjusted to 4.0 by adding NaOH and HCl; an arc light source equipped with a 350 W mercury lamp was used as the light source.
